# Automatic Graph Cut Segmentation of Lesions in CT Using Mean Shift Superpixels

**DOI:** 10.1155/2010/983963

**Published:** 2010-10-28

**Authors:** Xujiong Ye, Gareth Beddoe, Greg Slabaugh

**Affiliations:** R&D Department, Medicsight PLC, 66 Hammersmith Road, London W14 8UD, UK

## Abstract

This paper presents a new, automatic method of accurately extracting lesions from CT data. It first determines, at each voxel, a five-dimensional (5D) feature vector that contains intensity, shape index, and 3D spatial location. Then, nonparametric mean shift clustering forms superpixels from these 5D features, resulting in an oversegmentation of the image. Finally, a graph cut algorithm groups the superpixels using a novel energy formulation that incorporates shape, intensity, and spatial features. The mean shift superpixels increase the robustness of the result while reducing the computation time. We assume that the lesion is part spherical, resulting in high shape index values in a part of the lesion. From these spherical subregions, foreground and background seeds for the graph cut segmentation can be automatically obtained. The proposed method has been evaluated on a clinical CT dataset. Visual inspection on different types of lesions (lung nodules and colonic polyps), as well as a quantitative evaluation on 101 solid and 80 GGO nodules, both demonstrate the potential of the proposed method. The joint spatial-intensity-shape features provide a powerful cue for successful segmentation of lesions adjacent to structures of similar intensity but different shape, as well as lesions exhibiting partial volume effect.

## 1. Introduction

Accurate and automatic segmentation of medical images is an essential component of a computer-aided diagnosis (CADx) system. However, medical image segmentation is typically a difficult task due to noise resulting from the image acquisition process, irregular shape and variable size of anatomical objects, as well as the characteristics of the object's neighborhood. For example, a lung nodule or a colon polyp is usually embedded in a complex surrounding region. In CT imaging, the intensities of such lesions (e.g., juxtavascular nodules, juxtapleural nodules, or colonic polyps) are usually very similar to their adjacent tissues. In this case, traditional intensity-based or morphological methods [1–5] may fail to accurately segment the lesion. 

Energy minimization techniques for image segmentation have shown much promise in medical image computing. In particular, graph cut methods [6–12], which construct an image-based graph and achieve a global minimum of energy functions are often found in image segmentation. Two key issues in the graph cut technique are as follows: (1) how to define an energy function so its minimization leads to the desired result; (2) how to represent the energy function in terms of the graph construction. 

In most graph cut methods, the graph vertices are constructed at the image pixels, and the segmentation energy is composed of intensity terms only. For example, Zheng et al. [7] proposed a framework to simultaneously segment and register the lungs and nodules in CT data. For segmentation, a 2D pixel-based graph cut algorithm was applied to 3D lung and nodule datasets. It is noted that, when a graph vertex is placed at each image pixel, the number of nodes in the graph increases exponentially with the image size, dramatically increasing the computation time. To improve the efficiency, Li et al. [8] introduced a graph built on a presegmented image using a watershed algorithm. However, their graph cut formulation is solely based on the image intensities. It is known that pixel intensity can be locally erroneous due to noise and other image acquisition issues, such as Partial Volume Effect (PVE). Also, in CT imaging, different nearby anatomies may have similar intensities. Xu et al. [9] presented a graph cuts-based active contour approach to object segmentation, which Slabaugh and Unal [10] extended by incorporating a shape prior. These methods show promising results, but require manual initialization and quantitative evaluation on different types of lesions which are not available. Liu et al. [11] applied ordering constraints into an energy smoothness term based on an initial labeling. A simple geometric shape prior was also incorporated in a graph cut segmentation. Zheng et al. [12] constructed a graph Laplacian matrix and solved linear equations for the estimation of Ground Glass Opacity (GGO) nodules in CT, with assistance from some manually drawn scribbles for which the opacity values are easy to determine manually. More general-purpose graph cut methods show much utility in image segmentation, however, are ideally suited to nonmedical images. Other medical imaging approaches apply to nodule segmentation but are evaluated only on very small CT datasets. Further, most available methods require user interaction to identify initial foreground and background seeds. 

The goal of this paper is to develop an automatic and robust superpixel-based graph cut method for accurate segmentation of different types of lesions in CT imaging including solid and GGO nodules, as well as colonic polyps. 

One of the original contributions of this paper is that our graph is built on mean-shift superpixels. In this paper, a *superpixel* is a connected set of pixels sharing similar properties. We obtain superpixels by using five-dimensional mean shift clustering that incorporates joint spatial, intensity, and shape (JSIS) features. The superpixels express the local structure of the data in the five-dimensional feature space. Mean shift clustering reduces the data variation, thereby helping to produce accurate segmentations. By connecting superpixels instead of pixels, the algorithm produces better results and improves speed significantly. 

A second original contribution is our energy function, which incorporates the image intensity and the shape feature into a Markov Random Field (MRF) minimized with graph cuts. In particular, the intensity and shape information appear in both our unary and pairwise terms. Compared to our previous work in [13], we have improved our unary term in the energy function by considering both image intensity and shape features and investigated the method on a relatively large dataset that contains different types of nodules, including juxtavascular, juxtapleural, as well as GGO. Moreover, the method has also been applied to colonic polyp segmentation. The experimental results, evaluated both visually and quantitatively, demonstrate high performance for CT lung nodule segmentation. Initial results on colon polyps demonstrate the generalization of the method to other anatomies. 

The paper is organized as follows: Section 2 describes the proposed algorithm, which covers five-dimensional mean shift clustering based on JSIS features (Section 2.1) and automatic graph cut segmentation of the mean shift JSIS superpixels (Section 2.2).  Section 3 presents our experimental results, followed by a discussion and future work in Section 4 and conclusion in Section 5.

## 2. Methodology

**Figure 1:**
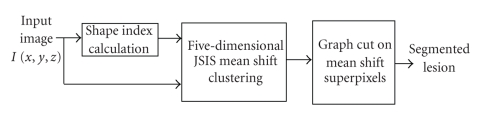
Flow diagram of the proposed graph cut-based method.

Our approach is a combination of five-dimensional mean shift clustering followed by energy minimization based on graph cuts. The method is also guided by prior knowledge about the lesion (e.g., nodule/polyp). The flow chart of our method is illustrated in Figure 1. In the following sections, each stage is described in detail.

### 2.1. Mean Shift Clustering of JSIS Features

The method first computes the JSIS features, which are then clustered in a five-dimensional space using mean shift. In this section, we review the volumetric shape index feature and our five-dimensional mean shift approach.

#### 2.1.1. Volumetric Shape Index: A 3D Geometric Feature

 The volumetric shape index (SI) at voxel *p* can be defined as follows [14, 15]:

(1) $$SI\left(p\right)=\frac{1}{2}-\frac{1}{\pi }\arctan\frac{k_{1}\left(p\right)+k_{2}\left(p\right)}{k_{1}\left(p\right)-k_{2}\left(p\right)},$$

where *k*_1_(*p*) and *k*_2_(*p*) are the principal curvatures, defined as

(2) $$\begin{array}{c} k_{1}\left(p\right)=H\left(p\right)+\sqrt{H^{2}\left(p\right)-K\left(p\right)},\\ k_{2}\left(p\right)=H\left(p\right)-\sqrt{H^{2}\left(p\right)-K\left(p\right)}, \end{array}$$

where *K*(*p*) and *H*(*p*) are the Gaussian and mean curvatures, respectively.

The calculation of the Gaussian and mean curvatures is based on the first and second fundamental forms of differential geometry. A practical approach for computing these forms is to use the smoothed first and second partial derivatives of the image as suggested in [16]. In this paper, prior to shape index calculation, a single-scale 3D Gaussian filter, with standard deviation of 1.5, is applied to obtain a smoothed image.

 The shape index provides a local shape feature at each voxel. Every distinct shape, except for the plane, corresponds to a unique shape index. For example, a shape index value of 1.0 indicates a sphere-like shape (e.g., lung nodule or colonic polyp), while 0.75 indicates a cylinder-like shape (e.g., vessel or colonic fold). Based on the definition, the volumetric shape index directly characterizes the topological shape of an isosurface in the vicinity of each voxel without explicitly calculating the isosurface. This feature provides rich information that complements the image intensity and is useful for automated segmentation. In particular, lesions in a CT image may appear within an area of complicated anatomy (such as a lung nodule neighboring a blood vessel or colonic polyp attached to the colon wall) where adjacent structures have similar image intensities but different shapes. 

 Note that we use the term *spherical concentration* throughout the paper to describe a spatially connected set of voxels that have a high shape index value.

#### 2.1.2. Inclusion of the Shape Index Feature in the Mean Shift Framework

 For each voxel, the 3D spatial location, intensity, and volumetric shape index features are concatenated in the joint spatial-intensity-shape domain of dimension *d* = 5. Given *n* data points *p*_*i*_, * i* = 1,…,*n* in a five-dimensional space, the multivariate kernel is defined as the product of three radially symmetric kernels

(3) $$K_{h_{s}h_{r}h_{si}}\left(p\right)=c_{k,5}k\left({||\frac{p^{s}}{h_{s}}||}^{2}\right)\cdot k\left({||\frac{p^{r}}{h_{r}}||}^{2}\right)\cdot k\left({||\frac{p^{si}}{h_{si}}||}^{2}\right),$$

where *c*_*k*,5_ is a normalization constant which assures that *k(p)* integrates to 1. *p* ^*s*^, *p* ^*r*^, and *p* ^*si*^ are the spatial location, intensity, and shape index features, associated with each voxel *p*; *h*_*s*_, *h*_*r*_, and *h*_*si*_ are the kernel bandwidths for spatial, intensity, and shape index kernel functions. The normal kernel is used in this paper, where *k*(*p*) = (2*π*)^−*d*/2^exp (−1/2||*p*||^2^). 

In the mean shift framework [17], the shape index feature can be combined with the intensity and spatial features for clustering. The mean shift vector with three kernel bandwidths (spatial, intensity, and shape index) is defined as

(4) $$\begin{array}{cc} & m_{h_{s}h_{r}h_{si}}\left(p\right)\\ & \;=\frac{\sum_{i=1}^{n}p_{i}g\left({||p_{i}^{s}/h_{s}||}^{2}\right)\;\cdot g\left({||p_{i}^{r}/h_{r}||}^{2}\right)\cdot g\left({||p_{i}^{si}/h_{si}||}^{2}\right)}{\sum_{i=1}^{n}g\left({||p_{i}^{s}/h_{s}||}^{2}\right)\;\cdot g\left({||p_{i}^{r}/h_{r}||}^{2}\right)\cdot g\left({||p_{i}^{si}/h_{si}||}^{2}\right)}-p, \end{array}$$

where *g*(*p*) = −*k*′(*p*). The mean shift vector always points in the direction of the maximum increase in the density function.

**Figure 2:**
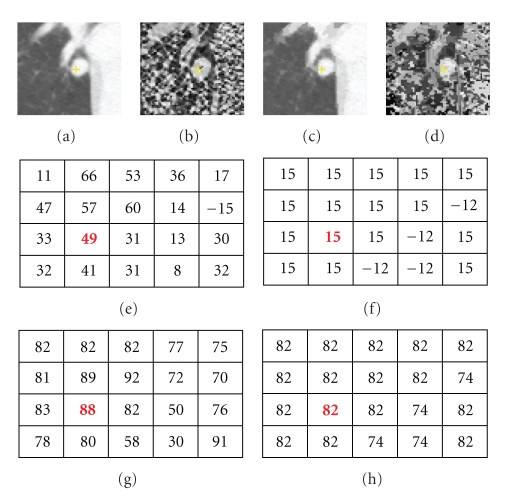
An attached nodule with its intensity and shape mode maps. (a) Original CT subimage; (b) shape index map based on (1); (c) and (d) intensity mode and shape index mode maps; (e)–(h) intensity values, intensity mode values, shape index values, and shape index mode values at the same voxel in the nodule. (It is noted that shape index are values multiplied by 100.)

It is noted that the mean shift algorithm estimates the *modes* (the densest regions) of the multivariate distribution underlying the feature space. The set of points that converge to the same mode is defined as the attraction basin. Mean shift maps all the data samples to the local maxima of their corresponding attraction basin based on five-dimensional features. *Superpixels* are formed for the set of pixels in each attraction basin. Each superpixel has a constant shape index and intensity, represented with *mode maps*, namely, an intensity mode map (*M*_*I*_) and a shape index mode map (*M*_*SI*_). Additionally, there is a spatial mode map (*M*_*S*_) that is not incorporated directly in our energy function; however, spatial information is utilized when defining neighbors in our graph.  Figure 2 shows a nodule attached to a vessel and its corresponding intensity and shape index mode maps. It can be noted that the mode maps ((c) and (d)) from JSIS mean shift clustering can be seen as “filtered” images and are less contaminated by outliers. In the following section, graph cut-based segmentation is applied to these superpixels.

### 2.2. Automatic Graph Cut Segmentation Using JSIS Superpixels

The mode map obtained by the above JSIS mean shift technique expresses the local structure of the data in a given region of the feature space. The number of resulting superpixels depends on the kernel bandwidth and the data itself; a key advantage of the mean shift clustering is its ability to locally group regions of similar intensity and shape, reducing the data variance in superpixel.

The aim of this section is to group superpixels into two classes: foreground (lesion) and background (nonlesion). It is known that the image labeling problem can be formulated using an energy function in a Bayesian framework in the context of maximization a posteriori (MAP) and MRF theory and can be solved by energy minimization. The energy function includes both unary and pairwise terms, the latter providing smoothing by modeling the interaction between neighboring superpixels. In this section, the MAP-MRF is transformed to a graph cut problem. A novel energy formulation that incorporates shape and intensity in both unary and pairwise terms is defined on superpixels and minimized with graph cuts using the maxflow/mincut method [6].

Two key issues are addressed in the following subsections: (1) how to initialize the segmentation and (3) how to define the energy function for minimization.

#### 2.2.1. Initialization Based on Spherical Concentration

In this paper, we assume that a nodule (or polyp) is generally either spherical or has a local spherical concentration, while a blood vessel (or colonic fold) is usually oblong. The initial seeds for the graph cut are computed automatically based on a spherical concentration. 

A spherical concentration *ℜ*_*s*_ is defined, using hysteresis thresholding [16], as a 3D connected region that satisfies the following criteria.

- All voxels in the region have a shape index greater than or equal to a low threshold *T*_*l*_.
- At least one voxel in the region has a shape index greater than or equal to a high threshold *T*_*h*_.

Typical thresholds are in the range  *T*_*l*_ ∈ [0.8,0.9) and  *T*_*h*_ ∈ [0.9,1].

**Figure 3:**
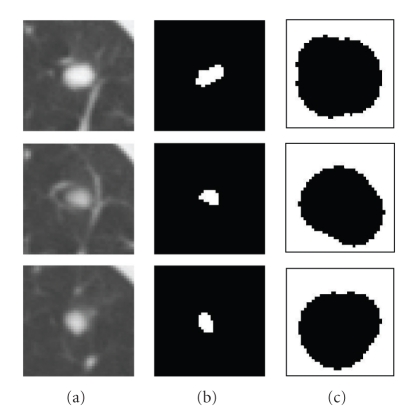
An example of the initialization based on spherical concentration on an attached nodule. (a) 3D nodule in three contiguous CT slices; (b) initial foreground based on high spherical concentration; (c) initial background region.

 The spherical concentration defines the foreground seeds in the graph cut initialization. The background seeds are determined using adaptive enlargement of the spherical concentration. A distance transform [18] of *ℜ*_*s*_ is computed and then adaptively enlarged (e.g., *μ*_factor_∗*f*DistanceMax, where *μ*_factor_ is the enlargement factor and *f*DistanceMax is the maximum distance to the region boundary). The background region can then be obtained by inverting this enlarged foreground region. The enlargement of the foreground region is designed so that the background region does not cover the foreground object.  Figure 3 shows an example of the initialization for a juxtavascular nodule using the above method, where *T*_*l*_ and *T*_*h*_ were set to 0.82 and 0.92, respectively. Figures 3(b) and 3(c) are the initial foreground and background regions, respectively, where *μ*_factor_ is set to be 10.

**Figure 4:**
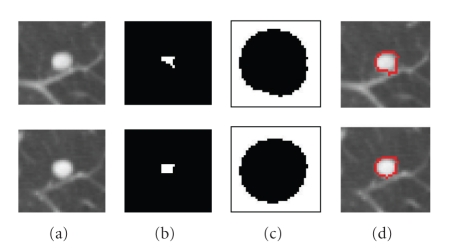
An example of the segmentation of an attached solid nodule based on the automatic calculation of the initial foreground and background. (a) 3D nodule in two contiguous CT slices; (b) initial foreground based on high spherical concentration; (c) initial background; (d) segmentation results by the proposed graph cut based method.

**Figure 5:**
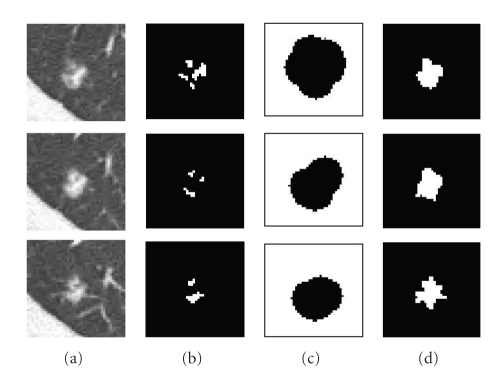
An example of the segmentation of a part-solid nodule based on the automatic calculation of the initialization (a) 3D nodule in three contiguous CT slices; (b) initial foreground based on high spherical concentration; (c) initial background; (d) segmentation results by the proposed graph cut based method.

 The thresholds of *T*_*l*_ and *T*_*h*_ are fixed throughout the paper and are based on the assumption that a nodule which may not be entirely spherical has at least spherical elements concentrated within the nodule. In fact, using shape index to extract the spherical concentration is the first step in our automatic lung nodule detection algorithm [19]. Very high detection sensitivity can be achieved at this stage. In most cases (e.g., high contrast solid nodule), the sphericity concentration covers the core part of the nodule. However, in the case of some part-solid or GGO nodules, the initial foreground region might spread in the nodule object. Figures 4 and 5 show examples of two different segmented nodules using automatic calculation of foreground and background regions, where, in Figure 4(b), the initial foreground contains the core part of the solid nodule object, while in Figure 5(b), the foreground region scatters in the part-solid nodule (it is noted, the foreground region is a 3D connected region but it may appear as several small regions in each 2D slice as shown in Figure 5(b)). In both cases, the proposed method provides better initialization. Figures 4(d) and 5(d) show the segmentation results by using the proposed graph cut-based method based on the initial foreground (b) and background seeds (c). However, occasionally the above initialization may fail to give good foreground and background seeds, as the spherical concentration may contain part of the nodule as well as lung tissue. An example is provided in Figure 21. More discussion for the initial foreground seeds will be given in Section 4.

#### 2.2.2. MAP-MRF Energy

In this section, we employ a graph *G* = (*V*, *E*), defined with vertices *v* ∈ *V* representing superpixels determined from five-dimensional mean shift clustering, and edges *ε* ∈ *E* connecting adjacent superpixels. A key difference from the usual graph construction is that we connect superpixels instead of the original pixels. As a result, the number of vertices in *G* is greatly reduced compared to the original number of pixels in the image.

 The lesion segmentation problem is formulated as a binary labeling problem, so the goal is to assign a unique label *l*_*i*_ ∈ {0,1} (where 0 is background and 1 represents foreground (lesion)) to each superpixel *i* by minimizing the following energy function *E*(*L*) [20]:

(5) $$E\left(L\right)=\sum_{i\in V}E_{1}\left(l_{i}\right)+\lambda \sum_{(i,j)\in \varepsilon }E_{2}\left(l_{i},l_{j}\right),$$

where *E*_1_(*l*_*i*_) is the unary data term representing the cost to assign the label *l*_*i*_ to the superpixel *i*, *E*_2_(*l*_*i*_, *l*_*j*_) is the pairwise smoothness term representing the cost of assigning the labels *l*_*i*_ and *l*_*j*_ to adjacent superpixels *i* and *j*, and *λ* is a weighting factor. The details of energy minimization via the graph cut algorithm for binary labeling can be found in [6]. Below we focus on how to define the two energy terms.

**Unary Data Term**

**Figure 6:**
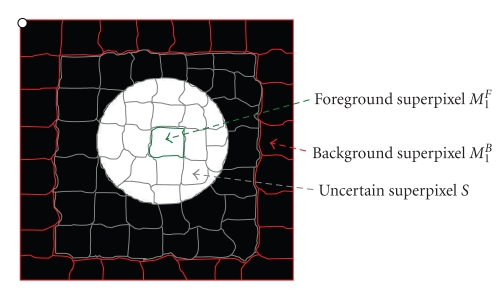
Schematic diagram of different types of superpixels: foreground (green) superpixels, background (red) superpixels, and uncertain (gray) superpixels.

We are given the initial foreground {*M*_*m*_^*F*^} and background regions{*M*_*t*_^*B*^}, automatically calculated from the previous section, where *m* and *t* are the superpixel indices for initial foreground and background, respectively.  Figure 6 shows the schematic diagram for different types of superpixels, for example, foreground, background, and uncertain superpixels. For each superpixel *i*, the unary term is defined as

(6) $$E_{1}\left(l_{i}=1\right)=\frac{c_{i}^{F}}{c_{i}^{F}+c_{i}^{B}},\;\;E_{1}\left(l_{i}=0\right)=\frac{c_{i}^{B}}{c_{i}^{F}+c_{i}^{B}},$$

where *c*_*i*_^*F*^ and *c*_*i*_^*B*^are the costs of a superpixel *i* to the foreground superpixels and background superpixels, determined from the initialization.

The foreground cost (*c*_*i*_^*F*^) is calculated as

(7) $$\begin{array}{cc} c_{i}^{F} & =\underset{m}{\min\;}\left\{1-\exp\;\left(-\frac{{\left(S_{I}\left(i\right)-M_{m}^{F}\right)}^{2}}{\sigma_{i}^{2}}\right)\right\}\\ & \;\times \left(1-\exp\;\left(-\frac{{\left(S_{SI}\left(i\right)-1.0\right)}^{2}}{\sigma_{si}^{2}}\right)\right), \end{array}$$

where *S*_*I*_(*i*) and *S*_*SI*_(*i*) are the intensity value and shape index value at superpixel *i*, determined from mean shift clustering. It is noted that the intensity and shape features are normalized to the same scale. Also, in this paper, the parameters *σ*_*i*_ and *σ*_*si*_ are determined experimentally (e.g., *σ*_*i*_ is chosen to be 0.6, while, *σ*_*si*_ is 0.3). It can be seen that *c*_*i*_^*F*^ encourages a superpixel to have the same label as the initial foreground superpixels if the superpixel has similar intensity to the foreground superpixels and also its shape feature is closer to one.

Similarly, the background cost *c*_*i*_^*B*^ is defined as

(8) $$\begin{array}{cc} c_{i}^{B} & =\underset{t}{\min\;}\left\{1-\exp\;\left(-\frac{{\left(S_{I}\left(i\right)-M_{t}^{B}\right)}^{2}}{\sigma_{i}^{2}}\right)\right\}\\ & \;\times \exp\;\left(-\frac{{\left(S_{SI}\left(i\right)-1.0\right)}^{2}}{\sigma_{si}^{2}}\right). \end{array}$$

**Pairwise Smoothing Term**

The second term *E*_2_(*l*_*i*_, *l*_*j*_) in (5) is defined as

(9) $$E_{2}\left(l_{i},l_{j}\right)=\left|l_{i}-l_{j}\right|\cdot \left(w_{si}\left(E_{I}+E_{SI}\right)+\left(1-w_{si}\right)\cdot E_{I}\cdot E_{SI}\right),$$

where *E*_*I*_ is an intensity energy term denoting the intensity difference between two adjacent superpixels *i* and *j*, defined as *E*_*I*_(*l*_*i*_, *l*_*j*_) = 1/(||*S*_*I*_(*i*) − *S*_*I*_(*j*)|| + 1). This means that superpixels with similar intensities have a larger *E*_*I*_, which leads to assigning them the same labels.

*E*_*SI*_ is the shape energy term denoting the shape difference between two adjacent superpixels, defined as *E*_*SI*_(*l*_*i*_, *l*_*j*_) = 1/(||*S*_*SI*_(*i*) − *S*_*SI*_(*j*)|| + 1). Similar to the intensity term, the shape energy term captures the shape features for the two adjacent superpixels. If adjacent superpixels have similar shape, *E*_*SI*_ is larger, with high probability, both superpixels have the same label.

As can be seen from (9), the intensity term and shape term are combined through a weighting function *w*_*si*_ which is defined as

(10) $$w_{si}\left(S_{SI}\left(i\right),S_{SI}\left(j\right)\right)=\exp\;\left(-{\left(\frac{S_{SI}\left(i\right)-S_{SI}\left(j\right)}{\sigma_{si}}\right)}^{2}\right).$$

**Figure 7:**
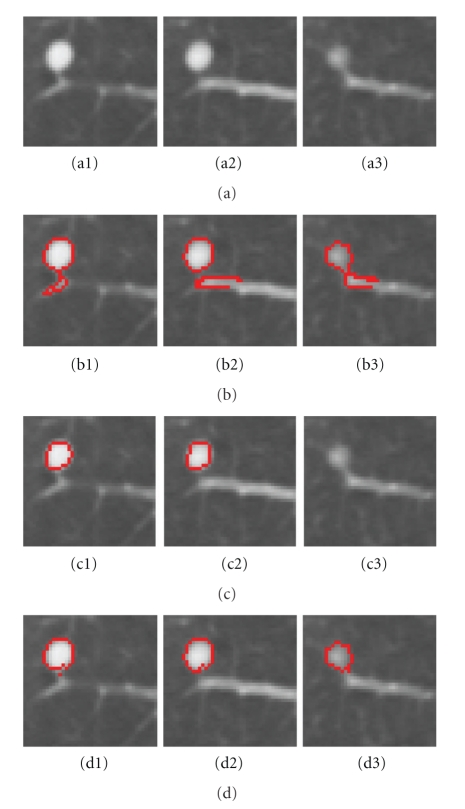
A example of an attached nodule segmentation using different pairwise smoothing energies. (a1–a3) 3D nodule in three contiguous slices in CT; (b1–b3) nodule segmentation using the first term only in (9); (c1–c3) results by using the second term only in (9); (d1–d3) nodule segmentation by using the complete energy defined in (9).

It is noted that when two adjacent superpixels have the same shape, namely, *w*_*si*_ = 1, the energy depends on the first term of (9). However, when two adjacent superpixels have very different shapes, *w*_*si*_ is small, so the (9) depends on the second term.  Figure 7 shows a juxtavascular nodule segmentation that uses different pairwise smoothing terms. Due to PVE in CT imaging, part of the nodule's pixels (e.g., Figure 7(a3)) has relatively low intensities, compared to those on other slices. With just the first term of (9), the pixels with low intensity (but similar shape) can still be correctly identified as being part of the nodule object, as seen in Figure 7(b3). However, some small amounts of vessels (which has similar intensity but a different shape feature value) are also included in the segmentation, as seen in Figures 7(b1–b3). This is because the first term equally considers the similarity for both of the intensity and shape features. Figures 7(c1–c3) are the results using the second term only in (9). It can be seen that the shape feature is only used as a weighting to the intensity feature. Superpixels with different shapes result in a lower weighting; this is why part of the nodule can be properly separately from the adjoining vessel despite the similar intensity, as shown in Figures 7(c1–c2). However, the superpixels with lower intensity due to PVE are wrongly identified as background due to the different intensities, compared to that on the other slices, as shown in Figure 7 (c3). Figures 7(d1–d3) are the results by combining both of terms as in (9), in which the nodule boundary can be properly delineated despite the PVE and the presence of vessels with similar intensities.

**Figure 8:**
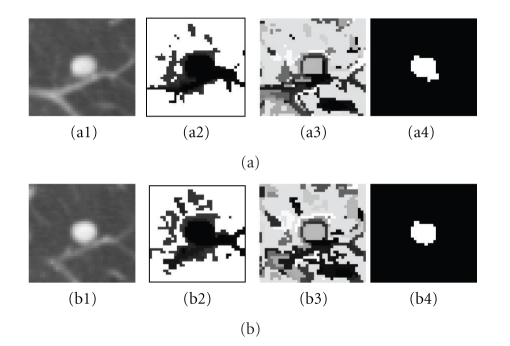
Example of cost functions. (a1, b1) 3D attached nodule in two contiguous slices in CT; (a2, b2) unary cost; (a3, b3) pairwise smoothing cost; (a4, b4) minimization of both energy terms based on (5).

Figure 8 shows a step-by-step example of using different cost functions for the energy minimization. Figures 8(a1) and 8(b1) are two contiguous slices for one 3D vascular nodule. Figures 8(a2) and 8(b2) are the unary cost (7) for each slice, respectively. The dark area in the image indicates lower cost for being foreground, while the bright area means high cost. Figures 8(a3) and 8(b3) are the pairwise smoothing term (9) for each slice, respectively. It can be seen that there are low costs for the boundary, while high costs are usually within the regions; Figures 8(a4) and 8(b4) are the minimization results in which the energy function uses both of unary term (6) and pairwise smoothing term (9).

## 3. Experimental Results

In this section, we present results demonstrating the effectiveness of the proposed algorithm applied to clinical CT scans. The evaluation of the proposed segmentation method has been conducted in two parts. The first, visual analysis is performed on several different types of CT pulmonary nodules and colonic polyps to provide insight into the method's performance for any visual gross missegmentation, such as a failure in separating nodules from vasculature. The second, quantitative experiments evaluate the segmentation results on large nodule datasets, containing 101 solid nodules and 80 GGO nodules. It is noted that, all the nodules in our database are confirmed by three experienced thoracic radiologists. However, each nodule boundary was manually delineated by one qualified radiologist.

 In all experiments, the three kernel bandwidths (spatial *h*_*s*_, intensity *h*_*r*_, and shape index *h*_*si*_ in (4)) in the five-dimensional JSIS mean shift clustering were set to 3.0, 6.5, and 3.0, respectively. The proposed graph cut algorithm was applied to the mean-shift superpixels using the energy formulation based on (5)–(10), where the weighting factor *λ* was set to be 1. The MRF parameters were selected manually based to maximize the overlap ratios with the ground truth data.

### 3.1. Comparison with Four-Dimensional Mean Shift and Graph Cut without the Shape Feature

#### 3.1.1. Lung Nodule Segmentation

**Attached Nodules**

**Figure 9:**
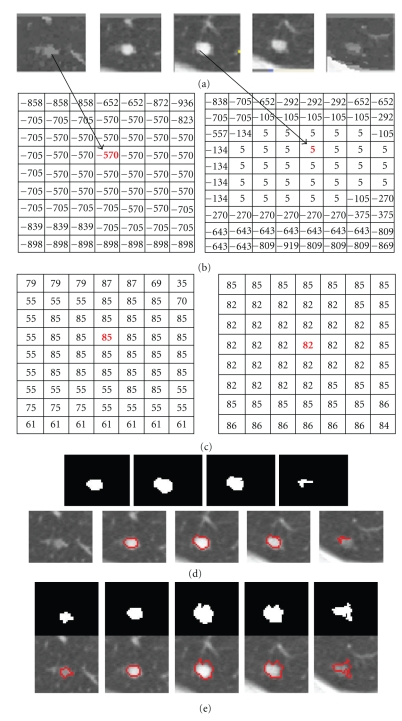
An example of a solid nodule (attached to a small vessel) segmentation. (a) Original 3D nodule on 5 contiguous slices; (b) and (c) intensity and shape index (multiplied by 100) mode maps from 5D mean shift clustering; (d) segmentation results without considering the shape feature; (e) results based on the proposed method.

Figure 9 demonstrates a detailed example of the segmentation of a solid nodule attached to a small vessel. The figure shows (a) the original CT lung nodule on five contiguous slices, (b), and (c) corresponding intensity mode and shape index mode values on two of the slices of the superpixels resulting from the five-dimensional JSIS mean shift clustering. Since each superpixel is an attraction basin in the 5D feature space, each superpixel has a different pair of intensity and shape index mode values. In Figure 9(b), after five-dimensional JSIS mean shift clustering, most of voxels within the nodule on the same slice have the same mode (such as the value of “−570” in intensity mode map of the first slice and “5” of the third slice). It can be seen that, although the intensity modes within nodule are very different, their shape modes are quite similar.

The nodule superpixels can be merged using the proposed graph cut method. Figure 9(d) shows the segmented nodule resulting from a graph cut without the shape feature, for which the intensity energy term *E*_*I*_ was only used in the pairwise term (9). The algorithm correctly segments most of the nodule (specifically, the part of nodule in the middle three slices). However, the other parts of the nodule with lower intensity (e.g., pixels on the first and the last slice) are wrongly identified as background due to the different intensities. Figure 9(e) shows the segmented nodule using the proposed method. It can be seen that, by combining both the intensity energy *E*_*I*_ and shape energy *E*_*SI*_ into the pairwise energy term (9), the parts of the nodule with low intensity can be successfully segmented due to the similar shapes on the first and last slices.

**Figure 10:**
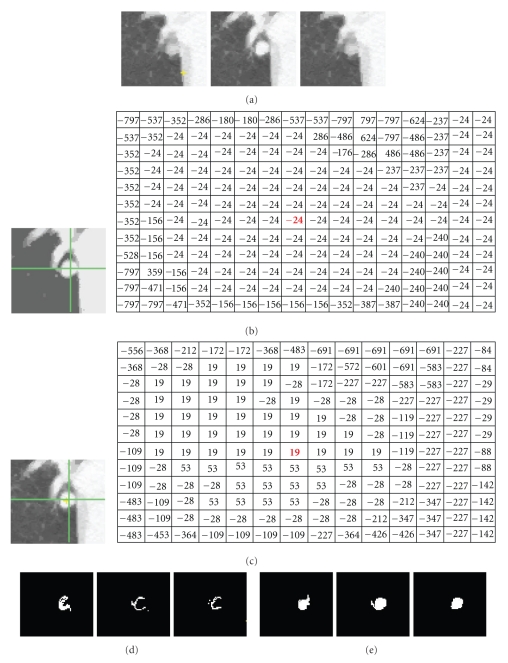
An example of segmentation of an attached nodule. (a) Original 3D nodule on three contiguous slices; (b) intensity mode map and its corresponding intensity mode values based on 4D mean shift clustering (without shape); (c) intensity mode map and its corresponding intensity mode values based on 5D mean shift clustering (with shape). (d) and (e) segmentation results without and with the shape feature (proposed method), respectively.

Figure 10 shows another example of a segmentation of an attached nodule. The intensity of the nodule is very similar to that of the adjoining vessel and heart wall. Figure 10(a) shows the nodule on three continuous CT slices; Figures 10(b), and 10(c) are two intensity mode maps corresponding to the same nodule slice produced by four-dimensional mean shift clustering (without the shape index feature) and five-dimensional mean shift clustering (with the shape index feature), respectively. It can be seen that, without considering the shape feature, a large amount of nonnodule voxels (e.g., heart) have a same superpixel as that of nodule voxels (e.g., “−24” in Figure 10(b)). However, using the proposed five-dimensional JSIS feature, most of the nonnodule object is separated from the nodule. This is due to the different local shapes of the nodule and the surrounding anatomy (such as the adjoining vessel and heart wall). The proposed five-dimensional mean shift clustering not only considers spatial and intensity information, but also shape features.  Figure 10(d) shows the final segmented nodule using graph cut on the above four-dimensional mean shift superpixels, for which only the intensity term is used in the energy function. It is noted that the superpixel with intensity mode value of “−24” in Figure 10(b) produced from four-dimensional mean shift without taking into account the shape feature contains part of the nodule but also nonnodule objects (such as heart). By representing each superpixel as a node in the graph construction and minimizing the energy function defined in (5), this superpixel is identified as nonnodule object. That is why most of the nodule voxels within this super pixel were wrongly identified as background (nonnodule object). For comparison, the segmentation results of the proposed method are shown in Figure 10(e). It is noted that, by using the proposed method, the misclassified nodule part in Figure 10(c) can be successfully identified as being part of nodule object.

**Pleural Nodule**

**Figure 11:**
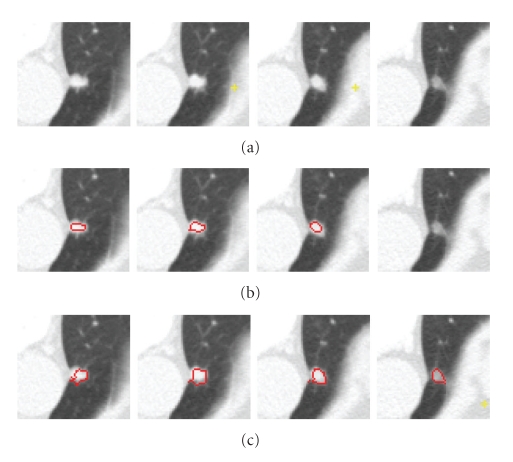
An example of a pleural nodule segmentation. (a) Original 3D nodule on four contiguous slices; (b) and (c) segmentation results without and with the shape feature (proposed method), respectively.

A pleural nodule is defined as a nodule attached to the pleural wall.  Figure 11 shows an example of a pleural nodule segmentation.  Figure 11(a) is a 3D nodule in four contiguous CT slices. Similar to the above two examples, Figures 11(b) and 11(c) compare results based on the 4D approach (no shape feature) to the 5D approach (using the shape feature). It can be seen that, without considering the shape feature, the algorithm is unable to segment the nodule on the last slice, while this missing part can be identified as part of the nodule by using our method.

**GGO Nodule**

**Figure 12:**
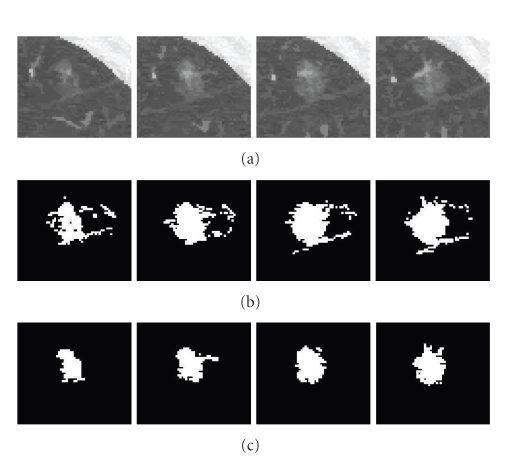
An example of a large GGO nodule segmentation. (a) Original 3D nodule on eight contiguous slices; (b) and (c) segmentation results without and with shape feature (proposed method), respectively.

A GGO nodule appears as a hazy increased CT attenuation of the lung. It is challenging to properly segment GGO boundaries due to the faint contrast, irregular shape, and fuzzy margins of GGO nodules.  Figure 12 shows an example of the five-dimensional method on a large GGO nodule (containing four slices as shown in Figure 12(a)), attached to small vessels with similar intensity. The segmentation results shown in Figure 12(c) demonstrate good performance of the method. For comparison, the results without the shape feature are also given in Figure 12(b). It can be seen that, without considering the shape feature, a large amount of small adjoining vessels are wrongly identified as part of the nodule object. However, using the shape index feature in both the mean shift clustering and the graph cut energy function, the nodule boundary can be properly delineated from the background despite the low intensity of the nodule and the presence of other nontarget structures with similar intensity.

Quantitative evaluation of GGO nodule segmentation and further discussion will be given in next subsection.

#### 3.1.2. Colonic Polyp Segmentation

**Figure 13:**
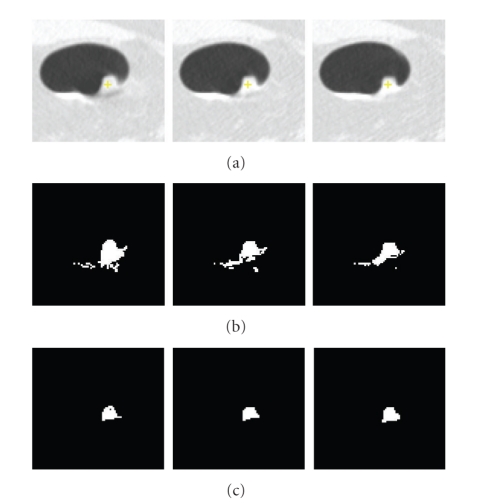
Segmentation of a colonic polyp. In (a), we show a 3D polyp in three contiguous slices, in (b) segmentation results without the shape feature, and in (c) segmentation results based on the proposed method.

**Figure 14:**
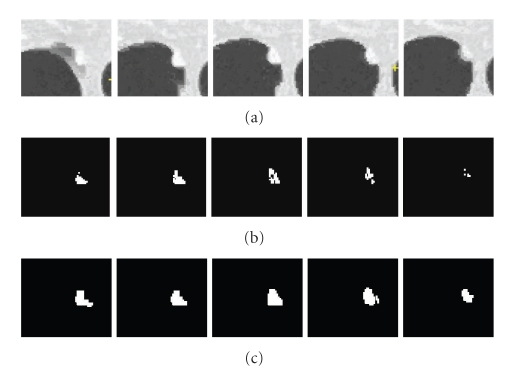
Another example of polyp segmentation. (a) 3D polyp in five contiguous slices; (b) segmentation results without shape feature; (c) segmentation results using the proposed method.

The segmentation of colonic polyps in CT images is a complex task due to the irregularity of polyp morphology and the complexity of surrounding regions. The boundaries between polyp tissues and nonpolyp tissues are much less obvious. Results showing the proposed method applied to colonic polyp segmentation appear in Figures 13 and 14. Similar to the nodule segmentation mentioned above, for comparison, Figures 13(b) and 14(b) show results without the shape feature, while Figures 13(c) and 14(c) are the results based on the proposed method. It can be seen that, by considering the shape index feature in both the mean shift clustering and the graph cut energy, both polyp boundaries can be properly delineated and separated from nonpolyp tissues. Both results demonstrate good performance of the proposed method for colonic polyp segmentation.

### 3.2. Comparison with Pixel-Based Graph Cut without Mean Shift Clustering

The performance of our superpixel-based graph cut algorithm was also compared with that of a standard pixel-based method, where the graph was constructed on each pixel and only the intensity was considered in the energy function.

**Figure 15:**
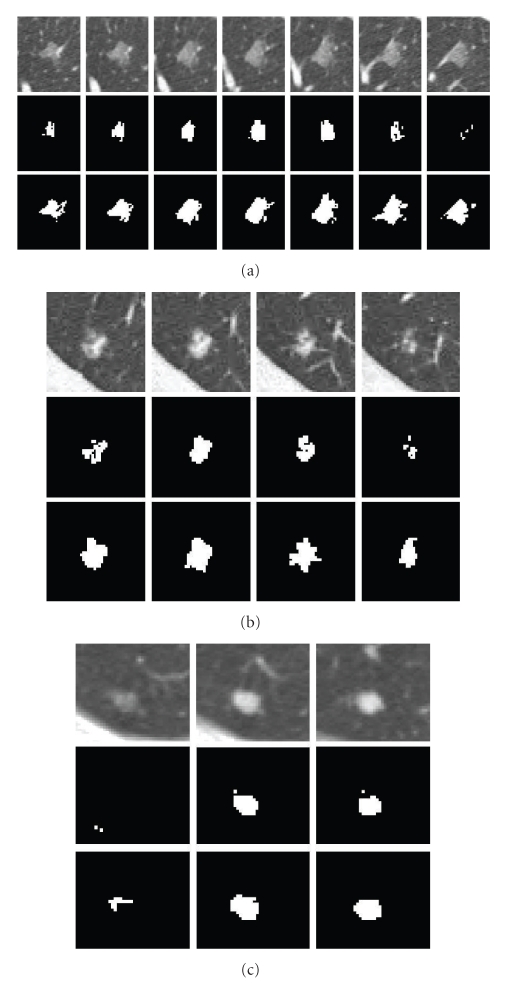
Comparison of pixel-based and superpixel-based graph cut algorithms on three different types of nodules. For each nodule in (a), (b), and (c), the first row shows the 3D nodule in the original CT subimages; the second row shows segmentation results based on the pixel-based method, and the last row shows results using the proposed superpixel based method.

Figure 15 shows segmentation results based on the two methods on three different nodules: (a) one large vascular GGO nodule, (b) one part-solid nodule, and (c) one solid nodule. Testing was performed on a system with a 2.39GHz CPU and 2GB memory. Table 1 shows the total number of vertices used in the graph cut algorithm and the computation time for both of pixel-based and superpixel-based methods. It is noted that the computation time in Table 1 includes construction of the graph and energy minimization. The majority of the computation time is in the graph construction, which includes the calculation of the both energy terms for each vertex. For the superpixel-based method, this also includes time for mean shift calculation. From Table 1, it can be seen that, on the superpixel-based graph, the fewer vertices result in a much faster run-time. Furthermore, from Figure 15, It is noted that on the pixel-based graph, only the core part of the nodule object is segmented while the outside part is wrongly labeled as a nonnodule object as shown in the middle row for each nodule Figures 15(a)–15(c). However, the proposed method shown in the third row for each nodule can correctly identify the nodules, separating nodules from the adjoining vessels.

Since the superpixels from the five-dimensional JSIS mean shift algorithm express the local structure of the data, it produces better results and improves the speed significantly.

### 3.3. Quantitative Evaluation

**Figure 16:**
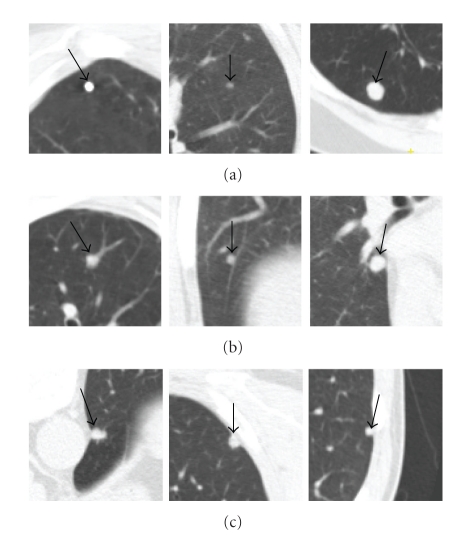
Example of different types of solid nodules. (a) Isolated nodules; (b) vascular nodules; (c) pleural nodules.

In this section, the proposed algorithm has been evaluated on a large set of 130 thoracic CT scans with a slice thickness ranging from 0.5 mm to 2.0 mm. The tube current ranged from 30 mA to 250 mA. Each scan was read individually by three experienced thoracic radiologists to produce a gold standard of 181 nodules (101 solid nodules and 80 GGO nodules). Among those solid nodules, 28 nodules are isolated nodule (in this paper, *isolated* nodule is a nodule not located near distracting structures of similar intensity, like large blood vessels, the pleural wall, etc.), 53 are vascular nodules, and another 20 are pleural nodules. Examples of different nodule types are shown in Figure 16. The size of the nodules ranged between 5 mm to 20 mm in diameter. To produce the ground truth, each nodule boundary was manually delineated by one qualified radiologist. 

Dice's coefficient (*R*) between each segmented nodule and the ground truth nodule is calculated as follows:

(11) $$R=\frac{2||V_{s}\cap V_{g}||}{||V_{s}||+||V_{g}||},$$

where *V*_*s*_ and *V*_*g*_ are the segmented nodule object and the ground truth object. The notation ||•|| gives the total number of voxels in the region, and the operator ∩ is an intersection.

#### 3.3.1. Evaluation on a Solid Nodule Dataset

**Figure 17:**
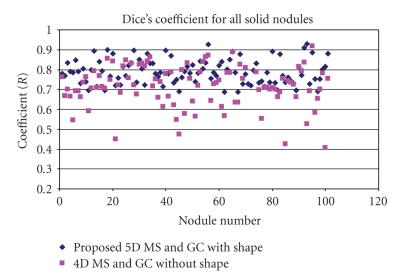
Dice coefficients based on the two different methods for all the solid nodules.

Figure 17 shows the overall distribution of the coefficients (*R*) calculated based on (11) for all solid nodules (101 nodules) using the proposed method and the method without the shape feature. It is noted that, without the shape feature, the mean Dice coefficient for the whole dataset is 0.71 with standard deviation (*σ*) of 0.1. However, using the proposed method, the mean Dice coefficient has been increased to 0.79 with the *σ* decreasing to 0.06. 

For comparison, we split all the solid nodules into three different types (isolated, vascular and pleural nodules) and evaluated the segmentation performance for each type separately.  Table 2 is the summary of Dice coefficients for each of the types of nodules, where the mean and standard deviation are calculated for the two different methods. 

For the isolated nodules, both methods give good results. For these nodules, the mean Dice coefficient for the 4D method is 0.80, and 0.81 for the 5D method. As can be seen in Figure 16(a), in general, isolated nodules have high contrast and well-defined boundaries, so the image intensity is the predominant feature, hence both methods obtain similarly good results. However, the proposed 5D method provides better results in the presence of PVE. In such cases, by considering the shape feature, the parts of the nodule with low intensity but similar shape can be successfully segmented. 

**Figure 18:**
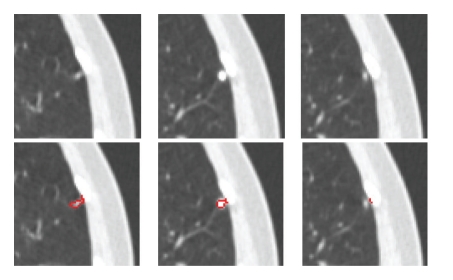
Example of a pleural nodule segmentation. Top row: original 3D nodule on three contiguous slices; bottom row: segmentation results based on the proposed method for which a small part of nodule on the last slice was incorrectly segmented.

**Figure 19:**
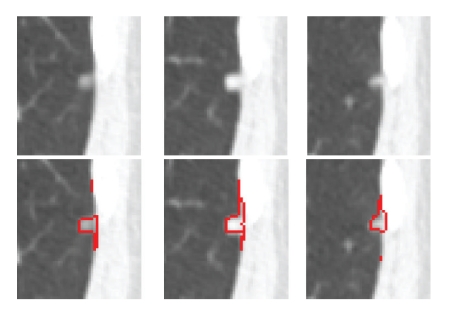
Another example of a pleural nodule segmentation. Top row: original 3D nodule on three contiguous slices; bottom row: segmentation results based on the proposed method in which the segmented nodule includes a small amount of lung wall.

 For both vascular nodules and pleural nodules, the proposed method gives a much better mean Dice coefficient. Especially for vascular nodules, the mean Dice coefficient increased from 0.68 to 0.8 with *σ* decreasing from 0.1 to 0.06 using the proposed method. In general, vascular nodule is embedded in a complex surrounding anatomy with similar intensities, and considering the shape feature provides rich information for the accurate segmentation. For pleural nodules, the mean Dice coefficient is 0.73 for the proposed method and 0.67 without the shape feature. Among these 20 pleural nodules, the best Dice coefficient is 0.88 by using the proposed method. Example of good segmentation of pleural nodule can be seen in Figure 11. However, Figure 18 shows an example of a small pleural nodule segmentation based on the proposed method, where the segmented nodule missed a small part of the nodule on the last slice, and Figure 19 shows a case where the segmented nodule boundary includes a small amount of lung wall tissue. More discussion for the pleural nodule segmentation will be given in next section.

#### 3.3.2. Evaluation on a GGO Nodule Dataset

**Figure 20:**
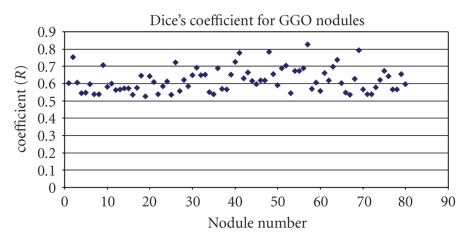
Dice coefficients for GGO nodules based on the proposed method.

The proposed method has also been evaluated on 80 GGO nodules (with pure and part opacified components). Most GGO nodules exhibit low contrast, irregular shapes and are often attached to vessels with very similar intensities.  Figure 20 shows the overall distribution of the Dice coefficients computed between each segmented GGO nodule and the ground truth, based on the proposed method. The mean overlap ratio is 0.63, with *std* of 0.07. The best Dice coefficient is 0.83, while the worst is 0.53.

GGO nodules pose a more difficult segmentation challenge than solid nodules. The proposed method provides a robust approach that combines both of local shape features and intensity. More discussion for GGO nodule segmentation will be provided in the next section.

## 4. Further Discussion and Future work

Experimental results presented in the previous section demonstrate the promise and generalization of the proposed method to different types of lung nodules in CT. Most of nodules can be properly delineated from the lung parenchyma despite the presence of other nontarget structures such as vessels or the lung wall. 

Based on the quantitative evaluation shown in Table 2, the mean Dice coefficient of the proposed method for both isolated nodules (0.81) and vascular nodules (0.8) is on average higher than that of pleural nodules. Moreover, for the vascular and pleural nodules, the mean Dice coefficient has been greatly improved when considering the shape feature, compared to without shape feature. Figures 18 and 19 show two small pleural nodules for which the proposed method either missed a small amount of nodule or included a small amount of adjoining tissues. We further investigated these cases for probable causes. In the mean shift clustering step, different fixed bandwidths are used in (3) for both the intensity and shape features, respectively. However, a fixed bandwidth might not be ideal, as a bandwidth that is too large might create superpixels that are too large, grouping small differences in intensity or shape that should otherwise be ungrouped. This can lead extraneous parts included in the final segmentation. To improve the performance, variable bandwidths for mean shift clustering will be further investigated. 

Figures 12 and 15(a) show our visual results on two GGO nodules, and Figure 20 provides a quantitative measure on 80 GGO nodules. The mean Dice coefficient of GGO nodules is, as expected, relatively lower compared to that of solid nodules. However, it is more difficult to segment GGO nodules due to the large variations in the intensities and vague boundaries. As an initial study, both in terms of a visual analysis and a quantitative evaluation on a relatively large dataset, the results are encouraging for accurate segmentation of GGO nodules in CT images. In general, GGO nodule has shown certain opacity pattern as shown in Figures 12 or  15(a); texture features such as grey level cooccurrence (GLCM) can be incorporated into mean shift clustering for the further improvement of the GGO segmentation. 

**Figure 21:**
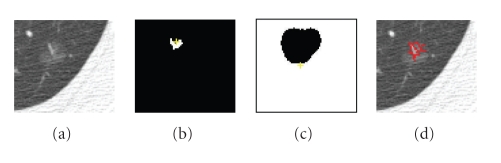
Segmentation results on a GGO nodule; (a) one slice of the GGO nodule; (b) initial foreground seeds located on the top border of the nodule; (c) initial background; (d) segmented nodule which is missing parts of the opacified component.

Also, in this paper, the initial foreground seeds for graph cut segmentation are automatically obtained based on spherical concentration. For our data, the initialization, described in Section 3, never failed completely, that is, the foreground initialization always returned some seeds within the nodule object for subsequent processing. In most cases, the initialization provided good foreground and background seeds for the graph construction and cut. The concentration of the high shape index values can either cover the core part of the object (e.g., solid nodules) or scatter within the nodule (e.g., some part-solid or GGO nodules). However, in the case of some part-solid or GGO nodules, the spherical concentration might be on the nodule boundary, which contains part of the nodule as well as the lung tissue. An example can be seen in Figure 21, where the initial foreground is on the top of the nodule border that contains some nonnodule parts, while the automatically obtained initial background also contains part of the nodule object. That can lead to the final segmentation missing part of the lesion. To resolve this issue, we are investigating a robust method which not only considers the shape feature but also intensity for the automatic generation initial seeds.

The proposed method has also been applied to colonic polyp segmentation. Visual analysis (Figures 13 and 14) shows the method's promise. The polyp boundaries can be accurately delineated from the adjoining nonpolyp tissues with very similar intensity. However, further analysis on a large dataset with different types of colonic polyps is required to test the generalization of the proposed method on this anatomy. This is one of the directions we will investigate in the near future.

## 5. Conclusion

We have presented a new, automatic method of extracting lesions from CT data. A five-dimensional JSIS mean shift clustering is firstly used to produce superpixels comprised of intensity and shape index mode maps. A graph cut algorithm is then applied using a novel energy formulation that considers not only image intensity but also shape. The initial foreground and background can be automatically obtained based on shape index concentration. The JSIS features provide rich information for lesion segmentation. Both by visual inspection on different types of lesions (lung nodules and colonic polyps), as well as using a quantitative evaluation on 101 solid nodules and 80 GGO nodules, demonstrate the potential of the proposed method. The method can not only successfully segment lesions adjacent to structures of similar intensity but different shape, but also can correctly identify some part of lesions with different intensity (due to PVE in CT imaging) but similar shape. 

## Figures and Tables

**Table 1:** Comparison of pixel-based and superpixel-based graph cut algorithms on three different types nodules.

Nodule type	Pixel-based method		superpixel-based method	
	Number of vertices	Time(s)	Number of vertices	Time(s)
GGO (a)	46195	16	946	1.2
part-solid (b)	22740	6.6	409	0.6
Solid (c)	17254	3.4	208	0.5

**Table 2:** Summary of Dice coefficients for different types of solid nodules based on two different methods.

Type	Number nodules	4D-based method		Proposed method	
		Mean coefficient	Std (*σ*)	Mean coefficient	Std (*σ*)
Isolated	28	0.80	0.05	0.81	0.04
Vascular	53	0.68	0.1	0.80	0.06
Pleural	20	0.67	0.09	0.73	0.06
